# Ultra‐High Friction and Adhesion in Hydrogel Layer Driven by Wet‐to‐Dry Transition Dynamics

**DOI:** 10.1002/advs.202507827

**Published:** 2025-07-12

**Authors:** Chenxu Liu, Tianhui Sun, Wenqing Chen, Hongjian Zhang, Pan Huang, Lin Yang, Yuan Yao, Qiongyao Peng, Ying Hu, Yonggang Meng, Yu Tian, Hongbo Zeng

**Affiliations:** ^1^ Department of Chemical and Materials Engineering University of Alberta Edmonton T6G 1H9 Canada; ^2^ State Key Laboratory of Tribology in Advanced Equipment Tsinghua University Beijing 100084 China; ^3^ Heavy Machinery Engineering Research Center of Education Ministry Taiyuan University of Science and Technology Taiyuan 030024 China

**Keywords:** dehydration, friction, hydrogel layer, shrinkage, ultra‐strong adhesion

## Abstract

Hydrogels are well‐known for their antifriction and lubricating properties, particularly in hydrated environments, where their water‐rich polymer networks enable effective friction reduction. However, during the wet‐to‐dry transition, a critical phase is identified during which the microstructures of a polyacrylamide hydrogel layer undergo volumetric shrinkage, leading to extensive interfacial contact and enhanced intermolecular interactions at solid interfaces. This process leads to a sharp increase in friction and adhesion forces. Sliding friction tests show that under a 2 mN load, the shear force peaked at 115 mN, corresponding to a remarkably high friction coefficient of 57.5. By leveraging this wet‐to‐dry transition, strong object gripping is successfully achieved across a range of surfaces. Notably, the hydrogel layer exhibits a high adhesion strength of 3.48 MPa on glass and 3.64 MPa on Si substrates. These findings offer new insights into hydrogel active gripping technologies and provide promising implications for soft robotics and adhesive interfaces.

## Graphical abstract

This work reveals a critical wet‐to‐dry transition in polyacrylamide hydrogel layers that induces volumetric shrinkage, resulting in enhanced interfacial contact and dramatically increased friction and adhesion. Leveraging this transition enables strong, reversible gripping on diverse surfaces, offering new insights for hydrogel‐based gripping technologies, soft robotics, and high‐performance adhesive interfaces.

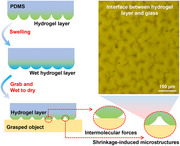

## 1 Introduction

Hydrogels are widely recognized for their exceptional lubrication properties and ultra‐low friction, making them valuable in various biomedical and engineering applications.^[^ ^1^, ^2^, ^3^, ^4^, ^5^ ^]^ Regardless of whether they exist in a dry or wet state or as layers or bulk materials, hydrogels typically exhibit a significant reduction in friction.^[^ ^6^, ^7^ ^]^ This behavior is primarily attributed to the lubrication provided by a hydrated polymer layer when the polymer chains are non‐adhesive to the substrate or the elastic deformation of adsorbed polymer chains when adhesion to the substrate occurs.^[^ ^8^, ^9^ ^]^ For example, in tribological studies, the friction coefficient (COF) of hydrogels typically ranges from 0.1 to 0.2 under dry conditions,^[^ ^6^, ^10^ ^]^ while under water‐lubricated conditions, it can be as low as 0.01 or even lower.^[^ ^1^, ^7^, ^11^ ^]^ Given these characteristics, hydrogels with excellent aqueous lubrication properties would seemingly have no apparent correlation with high friction, strong adhesion, or object gripping.

Through material modification, interfacial interaction optimization,^[^ ^12^, ^13^, ^14^ ^]^ and interlocking structure design,^[^ ^15^ ^]^ the adhesive strength of hydrogels can be significantly enhanced. However, conventional adhesive hydrogels often demonstrate limited adhesion performance, typically below 500 kPa. Expanding our scope of comparison, even the most recently reported transient semi‐glue tape utilizing water activation and self‐locking processes achieves adhesion strengths of 2.9 MPa on glass and 3.1 MPa on polyethylene terephthalate (PET) surfaces.^[^ ^16^ ^]^

If adhesion were solely governed by intermolecular interactions, achieving both high friction and strong adhesion with hydrophilic hydrogel materials would be challenging.^[^ ^17^ ^]^ For example, polyacrylamide (PAAm), a commonly used hydrogel,^[^ ^18^ ^]^ is generally not considered a viable adhesive material due to its inherently weak adhesion properties. However, recent observations indicate that hydrogel bulk materials or hydrogel layers undergo significant volumetric shrinkage during dehydration a phenomenon primarily driven by the collapse of the 3D crosslinked polymer network as water is expelled.^[^ ^19^, ^20^, ^21^, ^22^ ^]^ This structural transformation prompted our investigation into whether dehydration‐induced changes in the mechanical and adhesive properties of hydrogels could be exploited for novel applications. Notably, we discovered that PAAm hydrogel layers on polydimethylsiloxane (PDMS) substrate, which exhibit excellent lubrication in the hydrated or dry state, transition into a critical high‐viscosity phase during wet‐to‐dry transition process, leading to a dramatic increase in both friction and adhesion forces. The hydrogel layers developed in our study demonstrate an adhesion strength of 3.48 MPa on glass substrates, surpassing previously reported optimal values.^[^ ^16^ ^]^

To systematically explore this effect, we conducted low‐velocity unidirectional sliding friction experiments and found that, during the dehydration process, the COF of the hydrogel layer reached an exceptionally high value of 57.5. This phenomenon is attributed to the ultrahigh adhesion force generated during the transition from wet to dry, which enables robust object gripping. Unlike conventional adhesives that rely solely on intermolecular interactions, the strong adhesion observed here is driven by a combination of molecular interactions between the hydrogel layer and the gripped object, as well as the formation of special microstructures due to water loss and volumetric shrinkage of the hydrogel. These findings challenge the conventional understanding of hydrogels as solely lubricating materials and introduce a new perspective on their functional properties, particularly in the context of active gripping and adhesion‐based soft robotics.

## 2 Results and Discussion

### 2.1 Friction Behaviors of Hydrogel Layer during Wet‐to‐Dry Transition Process

In this study, a hydrogel layer composed of PAAm on a PDMS substrate was fabricated by an interfacial interpenetration strategy, involving utilizing hydrophobic initiators absorbed on the surface of polymer substrates and hydrophilic initiators in the hydrogel pregel solutions.^[^ ^6^, ^23^, ^24^ ^]^ The detailed fabrication processes can be found in the Method Section and Figure S1a (Supporting Information). The hydrogel layer refers to a coating composed of hydrogel formed on the surface of PDMS, with a thickness of ≈20 µm, featuring a 3D network structure at the nanoscale or submicron level (Figure S1b, Supporting Information).

**Figure 1:**
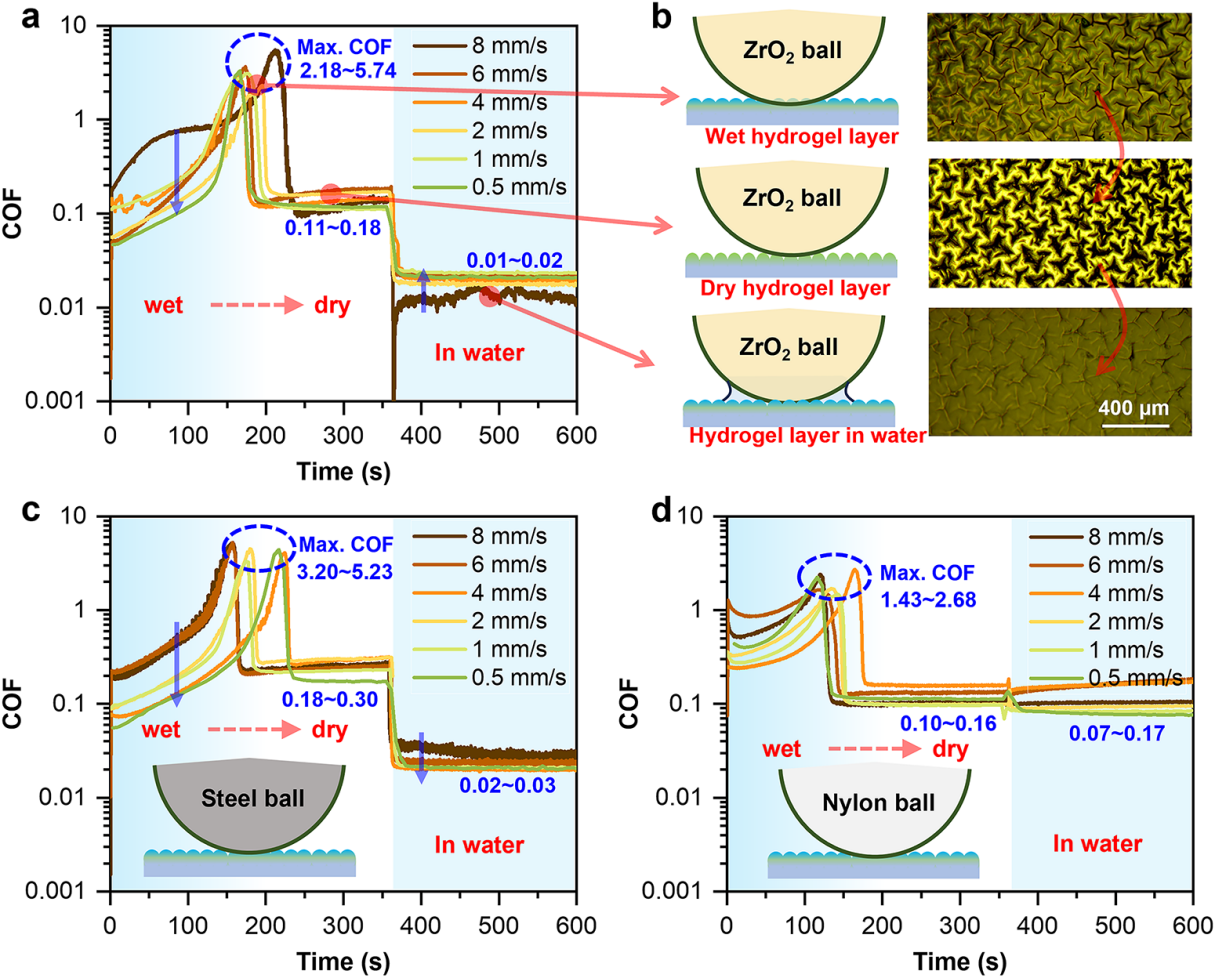
Reciprocating tribological behavior of the hydrogel layer under different conditions: a) COFs versus time when a ZrO₂ ball is used as the upper friction pair and the hydrogel layer as the lower friction pair, as the hydrogel surface transitions from a wet state to a dry state and subsequently to an underwater lubricated state; b) A schematic illustration of the friction system corresponding to (a) along with microscopic images of the hydrogel layer in its various states; c) COFs versus time when a stainless steel ball serves as the upper friction pair, showing the transition of the hydrogel layer from a wet state to a dry state and then to an underwater lubricated state; d) COFs versus time when a nylon ball is used as the upper friction pair, with the hydrogel layer undergoing the same sequence of state changes. In this experiment, the water on the surface of the hydrogel layer was gently removed with a non‐woven fabric at the start of the friction test to obtain a wet‐state hydrogel. Over a period of 100–250 s, the hydrogel layer gradually dried; then, at 360 s, ≈5 µL of DI water was added to induce underwater lubrication. The upper friction pair balls had a diameter of 2 mm, a normal load of 5 mN, a reciprocating stroke of 1 mm, and sliding velocities of 8, 6, 4, 2, 1, or 0.5 mm s^−1^.

After fabrication, the friction behaviors of the hydrogel layer under three distinct conditions, including wet, dry, and water‐lubricated states, were first tested. **Figure** **1**a presents the friction results obtained using a ZrO_2_ ball as the upper friction pair. Prior to initiating the friction tests, the surface water of the hydrogel layer was gently wiped away with a non‐woven fabric to achieve the wet state. After 100‐200 s, the hydrogel gradually dried, which was reflected in a steady increase in the COF—from an initial range of 0.04–0.20–2.18–5.74. Once the hydrogel layer was completely dry, the COF dropped markedly (to 0.11–0.18) and remained stable. At 360 s, ≈5 µL of deionized (DI) water was added to enable underwater lubrication, resulting in a COF of ≈0.01–0.02.

Figure 1b provides a schematic illustration of the friction system corresponding to Figure 1a, along with microscopic images of the hydrogel layer in its different states. The images show that the hydrogel surface is rough in the dry state, smooth under water, and intermediate in the wet state. Importantly, these three states are reversible, indicating that the COF can be modulated by controlling the surface condition of the hydrogel layer. In addition, microscopic observations (Figure S2, Supporting Information) of the hydrogel layer after the different stages of the friction process reveal that noticeable morphological changes occur in the wear region as the hydrogel transitions from a hydrated to a dry state and continues under dry friction. However, after rehydration and water lubrication, the surface morphology of this region nearly returns to its original state, making the wear track difficult to observe directly.

In the friction experiments described above, a normal load of 5 mN and a reciprocating stroke of 1 mm were applied. We further examined the effect of reciprocating velocities (0.5, 1, 2, 4, 6, and 8 mm s^−1^) on the COF. The experimental results revealed that, for the wet hydrogel layer, the COF tended to decrease with decreasing velocity; for the dry hydrogel layer, the COF exhibited no significant variation with velocity; whereas for the underwater hydrogel layer, the COF increased as the velocity decreased. A detailed explanation of the tribological mechanism will be provided in the following sections.

In addition to employing a ZrO_2_ ball as the upper friction pair, we also evaluated the frictional behavior of the hydrogel layer using other materials, including stainless steel and nylon balls, as illustrated in Figure 1c,d, respectively. The results indicate that, regardless of the upper friction pair material, the friction behavior and trends of the hydrogel layer under different conditions are fundamentally consistent. Specifically, the COF increases gradually during the transition from the wet to the dry state, with a pronounced drop observed ≈100–250 s.

**Figure 2:**
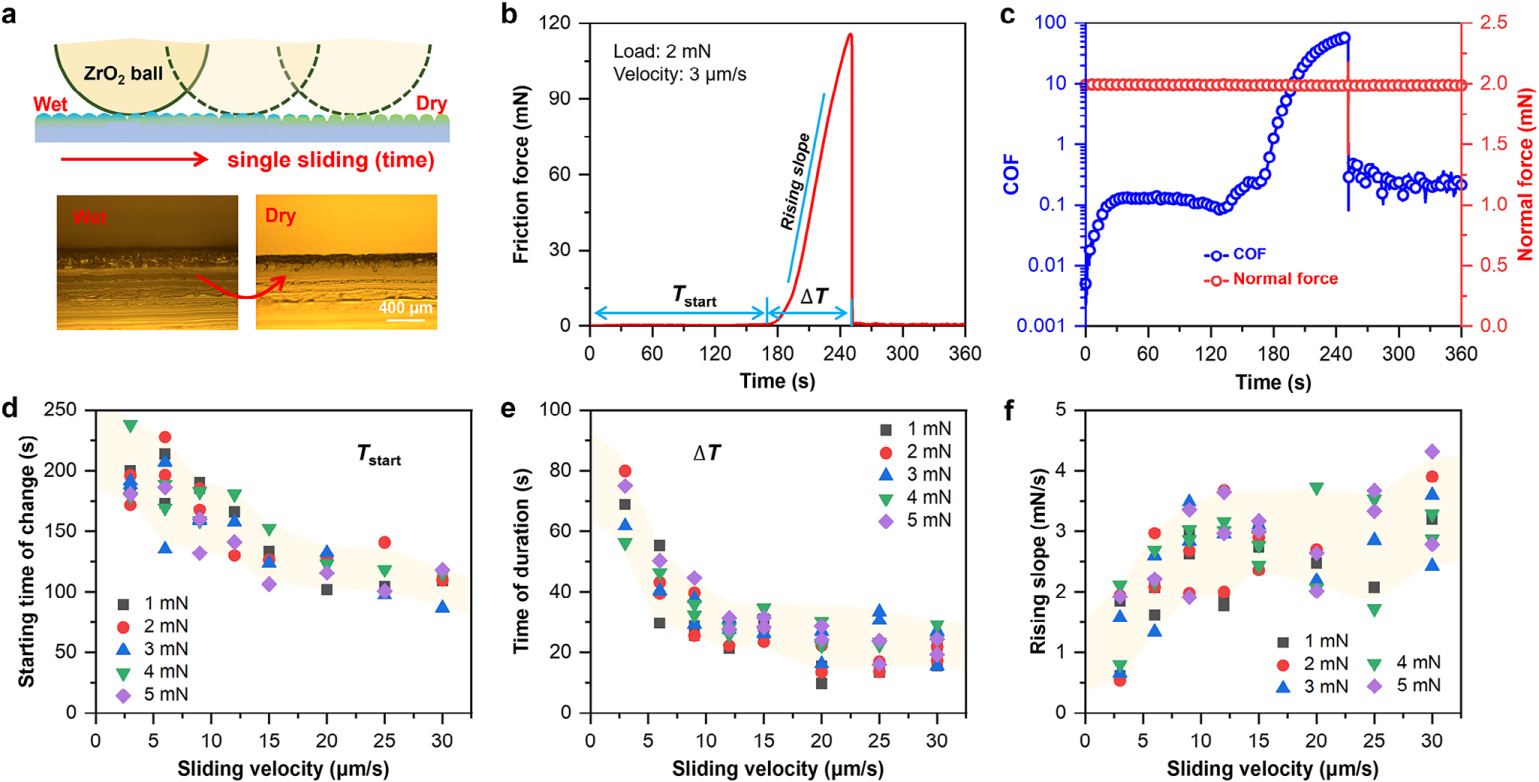
Design and results of a single sliding friction experiment. a) Schematic diagram of the single sliding friction test between the hydrogel layer against a ZrO_2_ ball, accompanied by cross‐sectional microscopic images illustrating the transition of the hydrogel layer from the wet to the dry state; b) Temporal variation of the COF during the single sliding process; c) COF and normal force variation over time under different loads; In the experiment, a ZrO_2_ ball with a diameter of 2 mm served as the upper friction pair, the applied load was 5 mN, the sliding velocity was 3 µm s^−1^, and the total sliding distance over 360 s was 1.08 mm; d) Onset time of the abrupt increase in friction force (*T*_start_) at different loads and velocities; e) Duration of the friction force increase (*ΔT*) at different loads and velocities; and f) Friction force rising slope at different loads and velocities. In these experiments, the upper friction pair was a ball with a diameter of 2 mm, the applied load ranged from 1 to 5 mN, the sliding velocity varied from 3 to 30 µm s^−1^, and the maximum sliding stroke was set to 5 mm.

Furthermore, a single sliding motion friction test was performed to analyze the COF and friction force of the hydrogel layer during its transition from a wet to a dry state at a low velocity of 3 µm s^−1^. **Figure** **2**a presents a schematic diagram of the single sliding friction test along with cross‐sectional microscopic images that illustrate the transition of the hydrogel layer from the wet state to the dry state. As the sliding time increases, the hydrogel surface gradually transitions from wet to dry, while the corresponding friction force is continuously recorded in real time under a certain normal load, as shown in Figure 2b and Figure S3 (Supporting Information).

The experimental results indicate that the thickness of the hydrogel layer in the wet state is similar to that in the dry state, both being ≈20 µm. However, the friction force begins to increase significantly at ≈170 s of sliding, rising at an almost constant rate from ≈0.2–0.6 to 115 mN within the subsequent 76 s. According to Figure 2c and Figure S4 (Supporting Information), when the load is between 2–20 mN, the change in normal force can be ignored during the transition of the hydrogel layer from wet to dry. Considering that the applied load is 2 mN (Figure 2c), the COF at this point reaches as high as 57.5. Almost simultaneously with reaching this peak, the friction force drops sharply to below 1 mN.

Based on the design of the aforementioned single sliding experiment, we further investigated the effects of varying the applied load (from 1 to 5 mN) and sliding velocity (from 3 to 30 µm s^−1^) on the onset time of the abrupt increase in friction force (*T*_start_), its duration (*ΔT*), the maximum friction force, and the friction force rising slope. The results indicate that *T*_start_ (Figure 2d), *ΔT* (Figure 2e), and the maximum friction force (Figure S5, Supporting Information) all decrease gradually as the sliding velocity increases. The structure of the hydrogel can adjust its interfacial interactions in response to changes in sliding velocity.^[^ ^25^, ^26^ ^]^ As a viscoelastic material, the hydrogel's energy dissipation is closely related to the sliding velocity. At low velocities, the polymer network within the hydrogel may exhibit delayed deformation, leading to greater energy dissipation and higher friction. In contrast, at high velocities, the hydrogel may display a more rapid strain recovery, which reduces internal energy dissipation and thus lowers the friction.

Notably, the influence of the applied load on these parameters is not significant. This can be attributed to the hydrogel's highly deformable nature; as the load increases, its structure adapts to the pressure by distributing the stress effectively. In addition, at low loads, the interface is likely in an adhesive friction state, wherein strong molecular interactions—such as hydrogen bonding and van der Waals forces—between the hydrogel's polymer chains and the ceramic surface result in higher friction. At higher loads, the hydrogel surface is highly susceptible to dehydration through poroelastic exudation, and the osmotic suction works against slip for higher friction.^[^ ^26^, ^27^ ^]^ Considering the combined effects of the factors, the effect of the load on friction is not significant under the conditions of this experiment. Figure 2f shows that the friction force rising slope tends to increase with sliding velocity, while the applied load does not have a significant effect on the rising slope.

### 2.2 Mechanical Characteristics of Hydrogel Layer during Dehydration

To verify the surface collapse caused by dehydration in the dry state and the swelling in the wet state of the hydrogel layer, we measured the surface of the hydrogel layer under different conditions using atomic force microscopy (AFM, Figure S6a,b, Supporting Information). The thickness of the loose outermost layer of the hydrogel (not the overall thickness of the hydrogel) was derived from the force curve detected as the AFM tip approached the surface.^[^ ^28^ ^]^ When the AFM tip contacts the hydrogel layer, it becomes surrounded or wetted by the soft surface. Consequently, capillary forces pull the tip toward the substrate until it reaches the firmer sublayer of the hydrogel. A similar capillary attraction occurs in the case of the hydrogel layer in the wet state, pulling the tip downward. Here, we use the jump distance as a measure of the loose outermost layer thickness.^[^ ^29^ ^]^ Due to the influence of van der Waals forces, this measurement serves only as an approximation.

**Figure 3:**
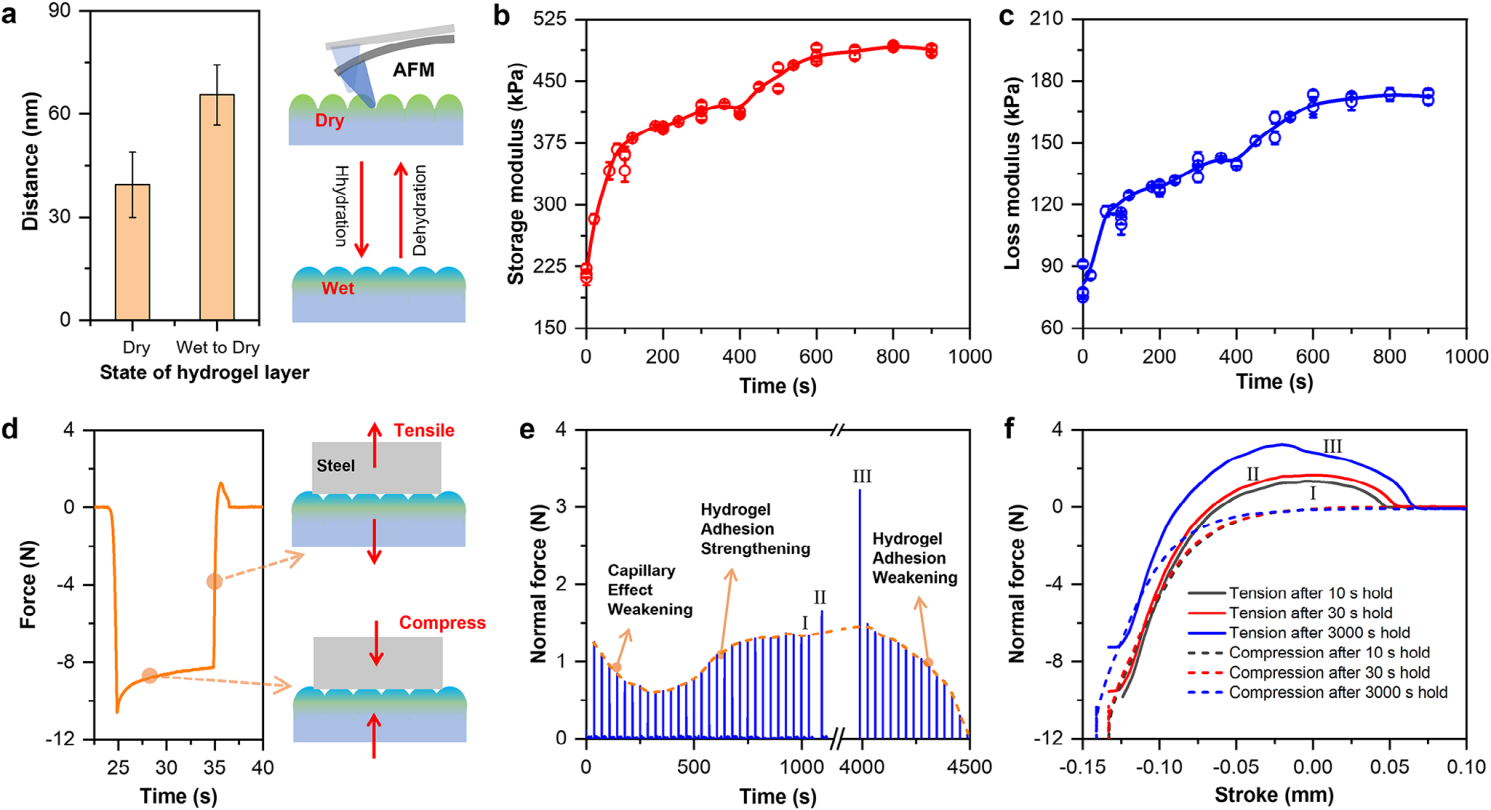
Mechanical properties of the hydrogel layer under various conditions: a) Thickness of the loose outermost layer of the hydrogel layer and structural model in both the dry state and during the transition from wet to dry. All tests were conducted in air at 21 °C with a relative humidity of 30–40%. The relationship curve of the storage modulus b) and loss modulus c) of the hydrogel layer on the PDMS surface with dehydration time (calculated based on the lifting and holding time). d) The applied force during one cycle of the macroscopic mechanical test; the test sample's area is ≈150 mm^2^; e) The variation of the adhesion force between the hydrogel layer and a stainless steel plate over time; f) Force‐displacement curves for the stretching and compression processes between the hydrogel layer and the stainless steel block after hold times of 10, 30, and 3000 s, respectively; The loading and stretching velocity for (d‐f) was 10 mm min^−1^.

By comparing the force curves in three different states (dry, wet, and during the transition from wet to dry), we obtained evidence that the hydrogel layer expands and increases in thickness from the dry state to the wet state. For the hydrogel layer in the dry state, the AFM tip moves a few nanometers under negative force until it reaches the substrate (Figure S6a, Supporting Information). In contrast, for the hydrogel layer in the wet state or during the wet‐to‐dry transition, the tip moves a greater distance under negative force due to the expansion of the surface crosslinked network structure (Figure S6b Supporting Information). As shown in **Figure** **3**a, the surface thickness of the hydrogel layer increases from 39.47 ± 9.50 nm in the dry state to 65.57 ± 8.74 nm in the wet‐to‐dry state. Furthermore, the adhesion force in the wet state or during the wet‐to‐dry transition is significantly higher than in the dry state, which is one of the reasons for the higher friction force in the former cases.

The relationship between the storage modulus and loss modulus of the hydrogel layer on the PDMS surface and the dehydration time (calculated based on the lifting and holding time) is shown in Figure 3b,c. At the initial stage of testing, the storage modulus and loss modulus of the wet hydrogel are 216.59 kPa and 81.24 kPa, respectively. During the gradual dehydration process, the storage modulus and loss modulus range approximately between 367.18–422.09 kPa and 113.34–142.68 kPa, respectively. When fully dried, the storage modulus and loss modulus reach 486.34 kPa and 171.61 kPa, respectively. The trend is consistent with the reported findings on the mechanical properties of hydrogels with different testing water contents.^[^ ^21^ ^]^

Macroscopic adhesion tests were also conducted. Figure 3d illustrates a single cycle of the test procedure. In this cycle, a load of ≈10 N was applied and maintained for 10 s; subsequently, the upper friction pair (a stainless steel plate) was lifted at a rate of 10 mm min^−1^ until a maximum displacement of 2 mm was reached. Immediately after reaching the peak, the friction pair was pressed downward at 10 mm min^−1^ and held under a load of 10 N for 10 s, and this cycle was repeated many times. As shown in Figure 3d, during the lifting phase, the normal force exhibited an upward increase at ≈35 s. This upward force is attributed to the adhesion force. Figure 3e presents the temporal variation of the adhesion force between the hydrogel layer and the smooth stainless steel plate. With increasing time, the capillary effect gradually diminishes while the adhesion force increases; as time continues to elapse and the capillary effect further weakens, the adhesion force subsequently decreases until it reaches zero. Similar phenomena were also observed in the adhesion tests between the hydrogel layer and glass plate, as well as ZrO_2_ plate (Figure S7, Supporting Information) under the same experimental conditions. However, in the case of nonwoven fabrics with high surface roughness, the initial behavior (capillary effect) is different.

Figure 3f displays the force‐displacement curves obtained during stretching and compression after hold times of 10, 30, and 3000 s, which correspond to processes I, II, and III in Figure 3e, respectively. The results indicate that the peak adhesion force increases with longer hold times (rising from 1.26  to 1.68 N, and further to 3.26 N). It is noteworthy that the durations in these tests are significantly longer than those in Figures 1 and 2. This is because the restricted space during the loading process inhibits the dehydration of the hydrogel layer.

### 2.3 Interface Observation of Hydrogel Layer during Dehydration

**Figure 4:**
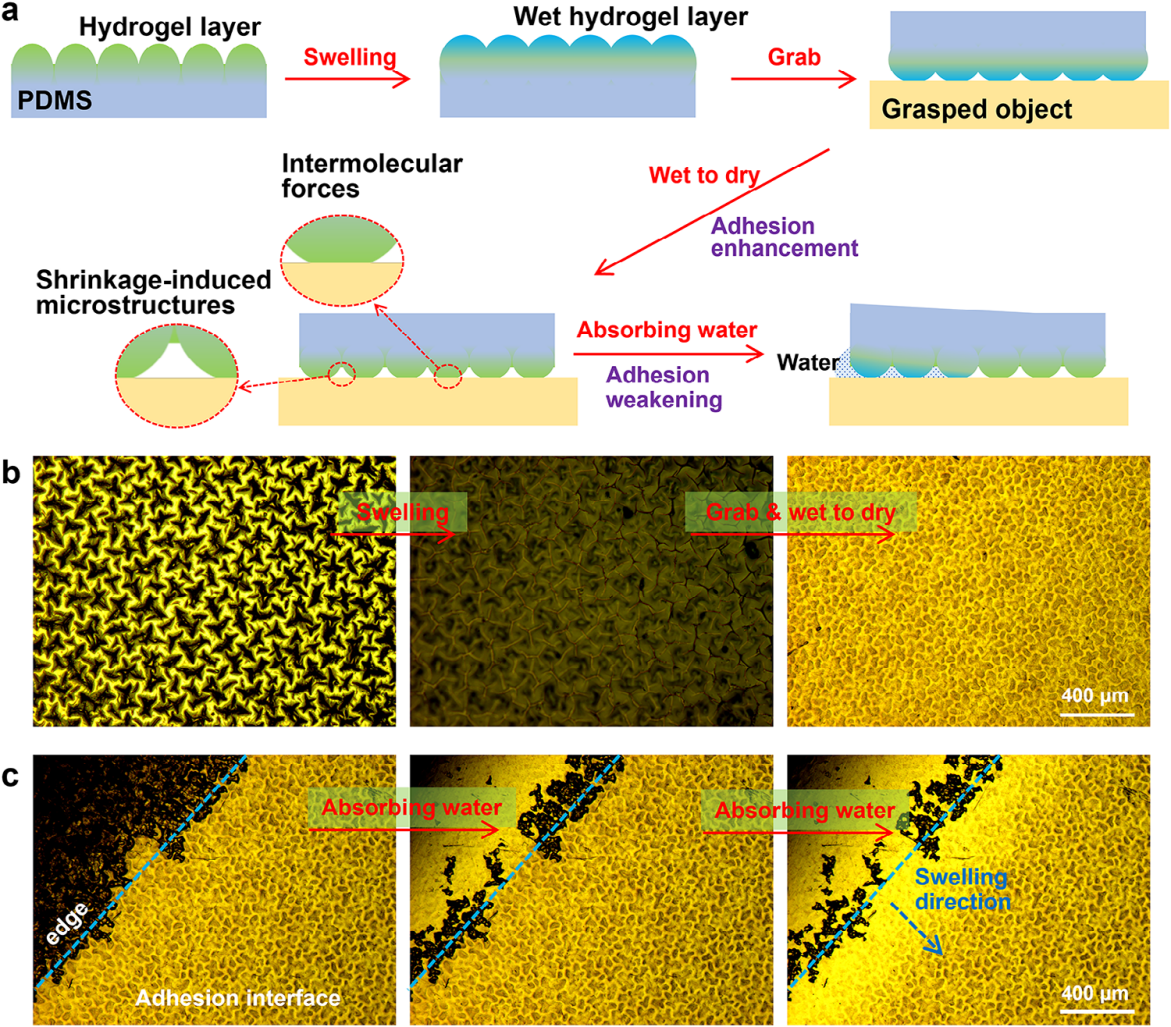
Schematic diagram and observation of the hydrogel layer grasping and release process. a) Schematic diagram of the process of using a hydrogel layer to grasp and release an object; b) Microscopic morphologies of the hydrogel layer in a dry state, a water‐absorbed swollen state, and in contact with a steel surface; In the third picture, the adhesion interface between the hydrogel and the target object was observed from the side of the PDMS substrate; c) Microscopic morphology of the contact interface between the hydrogel layer and the steel surface, and the changes during the water absorption process at its edge. The blue dashed curve in (c) indicates the boundary of the adhesive interface, while the black region represents areas where air is present between the hydrogel layer and the steel surface (non‐adhesive region).

**Figure** **4**a shows the schematic diagram of the process of using the hydrogel layer to grasp and release an object. First, the wet hydrogel layer is brought into contact with the object. During the transition of the hydrogel layer from wet to dry, a controlled pressure was applied to ensure intimate contact between the hydrogel layer and the surface of the target object. Once the hydrogel layer has dried, the adhesive force is significantly enhanced due to intermolecular interactions and the special microstructures formed by the contraction of the hydrogel layer's microprotrusions. Figure 4b shows the microscopic morphologies of the hydrogel layer in a dry state, a water‐absorbed swollen state, and in contact with the smooth surface of stainless steel.

The surface of the air‐dried hydrogel layer consists of wrinkles measuring 200–300 µm. After swelling, the surface roughness decreases, and the wrinkles become less pronounced. Upon further contact with a stainless steel surface and subsequent drying, the original wrinkles transform into circular or elliptical shapes with diameters of ≈100 µm. These structures were considered to be caused by contractile stress or negative pressure formed at the contact interface due to the dehydration of the hydrogel layer.

Figure 4c depicts the microscopic morphologies of the edge of the hydrogel layer in contact with the steel surface and the swelling changes of the hydrogel layer after water absorption. Through real‐time observation, we measured that the rate of water absorption at the interface of the hydrogel layer adhering to the steel surface starts from the edge at 0.5–1.0 µm s^−1^. As the adsorption time increases, the adsorption rate decreases linearly, as shown in Figure S8 (Supporting Information).

### 2.4 Adhesion Performance and Mechanism Analysis

**Figure 5:**
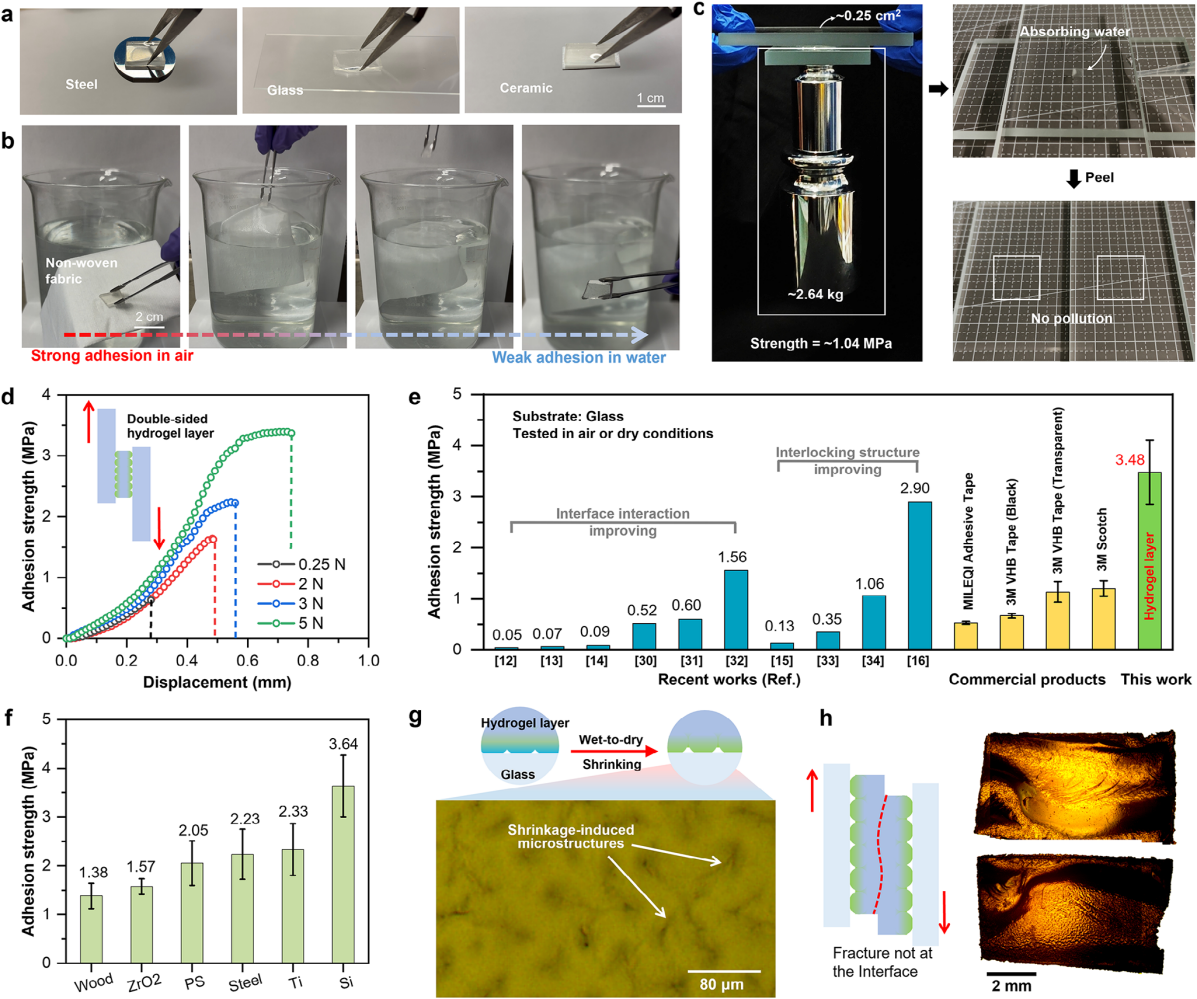
Adhesion performance testing, evaluation, and mechanism analysis of hydrogel layers. a) Photographs of real friction pairs demonstrating the use of a hydrogel layer as a gripper to capture stainless steel, glass, and ceramic plates; b) The case of the hydrogel layer gripping nonwoven fabric under different conditions, including in air and in water; c) Photographs of double‐sided hydrogel layer on glass plate and stainless steel plate sustaining the force of ≈2.64 kg stainless steel weight and the pollution‐free detachment. The contact area between the hydrogel layer and the glass plate is ≈0.25 cm^2^, with a calculated pressure of ≈1.04 MPa; d) The relationship between the adhesion strength and displacement measured in the adhesion test between the double‐sided hydrogel layer and glass plates under different pre‐loads. The stretching speed in adhesion test was 20 mm min^−1^; e) A comparison in adhesion strength of double‐sided hydrogel layer to various adhesives in recent researches and commercial products; f) Adhesion strength of double‐sided hydrogel layer on various substrates; g) Schematic diagram and microscopic observation image of the adhesion interface between the hydrogel layer and glass captured using reflected light; h) Microscopic observation image of the double‐sided hydrogel layer after the adhesion tests: Fracture occurs within the PDMS substrate rather than at the interface between the hydrogel layer and the glass.

The hydrogel layer demonstrates the ability to grip objects with a wide range of surface characteristics, including smooth, rough, and even fibrous surfaces. **Figure** **5**a depicts the use of a hydrogel layer as a mechanical gripper for capturing stainless steel, glass, and ceramic plates. To achieve a relatively high adhesion force, a wet hydrogel layer was pressed against the surfaces of these solid objects; after natural drying or heating at 75 °C, a strong adhesion was obtained, enabling the objects to be picked up. Figure 5b shows the case of the hydrogel layer grasping nonwoven fabric. In the air, the adhesion between the hydrogel layer and the non‐woven fabric fibers is strong. However, when submerged in water, the adhesion between the two significantly decreases, leading to their separation. This phenomenon is primarily attributed to the swelling of the hydrogel layer upon water uptake and the disruption of pre‐existing intermolecular forces at the adhesive interface, such as hydrogen bonding, caused by the presence of abundant free water molecules.

A weight‐lifting test was conducted to directly demonstrate the strength of the fabricated double‐sided hydrogel layer (Figure 5c; Movie S1, Supporting Information). Thick glass plates (0.14 kg weight) were used as both surfaces contact with the double‐sided hydrogel layer for reliable loading and bearing. The sample was cut to 0.5 cm × 0.5 cm in the dry state and then moistened with deionized water. After the sample turned into the wet state, it was carefully placed on one glass plate and covered by another, followed by applying a weight of 100 g as the preload. After complete drying at 75°C, one of the glass plates was adhered to the weights with commercial tape. The weights and glass plate, ≈ 2.64 kg in total, were manually lifted, showing the adhesion strength of ≈ 1.04 MPa. Moreover, pollution‐free detachment can be easily achieved by putting the sample into the wet state.

Figure 5d shows that the shear strength is related to the loading force applied during the sample preparation process. As the loading force increases from 0.25 to 5 N, the shear strength between the double‐sided hydrogel layer sample and the glass increases. This is related to the actual contact area between the hydrogel layers and the glass plates, which is influenced by the applied load (Figure S9, Supporting Information). The maximum shear adhesion strength between the double‐sided hydrogel layer and glass plates measured in this work reaches 3.48 ± 0.62 MPa. To further evaluate the adhesive performance of the hydrogel layers, normal (perpendicular) adhesion tests between the hydrogel and glass were also conducted. Experimental results showed that the normal adhesion strength was 3.51 ± 0.35 MPa, which is comparable to the shear adhesion strength.

For comparative analysis, we conducted shear adhesion strength tests under carefully controlled experimental conditions to ensure consistency, comparing our hydrogel layer with reported adhesives and commercial double‐sided tapes, as illustrated in Figure 5e. Based on adhesion mechanisms, these can be categorized into two primary classes: intermolecular interactions and pressure‐sensitive interlocking. The former encompasses various phenomena including the self‐hydrophobization process of hydrogel adhesives,^[^ ^12^ ^]^ interfacial hydrogen bond‐reinforced adhesion,^[^ ^13^ ^]^ dipole‐dipole interactions^[^ ^14^ ^]^ or ion‐dipole interactions^[^ ^30^ ^]^ between hydrogels and substrates, incorporation of boronic ester‐based dynamic covalent bonds into polymer networks,^[^ ^31^ ^]^ surface treatments to enhance surface free energy of polymethyl methacrylate (PMMA),^[^ ^32^ ^]^ and et al. However, most reported adhesion strengths under these mechanisms typically do not exceed 1.56 MPa.

Structural design strategies have demonstrated potential for enhancing adhesion performance. For instance, light‐ or heat‐initiated hydrogel shrinkage has enabled poly(N‐isopropylacrylamide) to reach 0.134 MPa adhesion strength on glass in the dry state.^[^ ^15^ ^]^ Through the development of supersaturated and stimuli‐responsive phase change hydrogel materials, adhesion strengths of up to 0.348 MPa can be achieved.^[^ ^33^ ^]^ Similarly, reversible transformation between soft and rigid states in electrothermal dry adhesives has achieved 1.063 MPa adhesion strength on glass under dry conditions.^[^ ^34^ ^]^ More recently, water activation and self‐locking processes have achieved remarkable adhesion strengths of 2.9 MPa on glass using transient semi‐glue tape.^[^ ^16^ ^]^

These strategies, to varying degrees, enhance interfacial interactions or mechanical interlocking at the bonding interface, resulting in significant improvements in adhesion performance. Notably, our hydrogel layer with shrinkage‐induced microstructures achieved an unprecedented adhesion strength of 3.48 MPa on glass, representing the highest reported value for adhesives. This value represents an ≈3–7 times enhancement in adhesion strength compared to commercial double‐sided adhesive tapes (Figure S10, Supporting Information).

A comprehensive evaluation of the adhesion performance of the hydrogel layer was conducted on multiple substrate materials, including polystyrene (PS), wood, silicon wafer (Si), titanium alloy (Ti), stainless steel, and zirconium dioxide (ZrO_2_). It is well established that adhesive materials typically exhibit substrate‐dependent performance characteristics due to variations in surface energy and hydrophobicity among different materials. The experimental results demonstrate the exceptional adaptability of the hydrogel layer, as shown in Figure 5f and Figure S11 (Supporting Information). The measured adhesion strength between the hydrogel layer and different substrates varied across materials. Specifically, the adhesion strength with steel and Ti plates was 2.23 ± 0.51 MPa and 2.33 ± 0.53 MPa, respectively, while for PS, it reached 2.05 ± 0.46 MPa. Most notably, the adhesion strength between the hydrogel layer and the Si wafer was as high as 3.64 ± 0.63 MPa, highlighting its superior adhesive performance.

In contrast, the adhesion strength between ZrO₂ ceramic plates and wood was comparatively lower, measuring ≈1.57 ± 0.16 MPa and 1.38 ± 0.26 MPa, respectively. This difference is primarily attributed to the rough surface of the ZrO_2_ ceramic plates as well as the wood plates, which reduces the actual contact area between the hydrogel layer and the substrate compared to the Si wafer or glass. Additionally, the bond strength between the nonwoven fabric and the single‐sided hydrogel layer is shown in Figure S12 (Supporting Information), with a measured peeling force per unit width of 235 ± 48 N/mm under 180° peeling.

Figure 5g and Figure S13 (Supporting Information) illustrate schematic diagrams of the adhesion interface between the hydrogel layer and various objects, accompanied by microscopic images captured using both reflected and/or transmitted light. In the case of glass object, the interface between the object and the hydrogel layer is characterized by numerous micro‐protrusions, aligning with the observations presented in Figure 4c. Microscopic analysis of the contact interface indicates that the strong adhesion is primarily driven by intermolecular interactions between the hydrogel layer and the gripped object, as well as the formation of the shrinkage microstructures resulting from water loss in the hydrogel. These structures function similarly to “negative pressure suction cups.”^[^ ^15^, ^35^, ^36^, ^37^ ^]^ Further analysis suggests that these formations arise from the contraction of the hydrogel layer due to water loss, creating an internal pressure significantly lower than the external atmospheric pressure. This mechanism enhances the adhesion strength between the object and the hydrogel layer. In addition, intermolecular interactions, particularly hydrogen bonding, also play a critical role in enhancing adhesion. The hydrogel layer used in this work is composed of PAAm chains containing amide groups (─CONH_2_), which feature both a hydrogen atom (H) bonded to nitrogen (N) and a carbonyl oxygen (C═O). These functional groups can act as both hydrogen bond donors and acceptors. Specifically, the H atoms in the amide groups can form hydrogen bonds with the oxygen atoms present in the SiO_2_ layer on the Si wafer surface, leading to strong interfacial adhesion between PAAm and the Si wafer. Similarly, the amide groups are also capable of forming hydrogen bonds with hydroxyl groups (─OH) present on the glass surface.

When interacting with a relatively rough ZrO_2_ plate, the interface with the hydrogel layer reveals irregular structures on the scale of several hundred micrometers (Figure S13b, Supporting Information). A similar phenomenon occurs when two hydrogel layers come into contact (Figure S13c, Supporting Information). In contrast, when the gripped object is nonwoven fabric, the fibrous nature of the material reduces the prominence of these special microstructures (Figure S13d, Supporting Information). Despite this, the adhesion remains relatively strong due to significant intermolecular interactions between the hydrogel layer and the nonwoven fabric, although it is anticipated to be weaker compared to the three scenarios previously described.

The hydrogel layer exhibits exceptionally strong adhesion to both glass and smooth steel surfaces, with the adhesive strength surpassing even the shear strength of the substrate material (PDMS). By observing the sample morphologies before and after the mechanical performance tests in Figure 5h and Figure S14 (Supporting Information), it can be concluded that the normal or shear adhesion strength between the hydrogel layer and the glass is greater than the strength of its substrate (the mechanical strength of the PDMS coated by hydrogel layer). In addition, infrared spectroscopy was performed on the residues remaining on the glass surface and compared with the original PDMS sample. The result (Figure S15, Supporting Information) revealed that the chemical composition of the fractured surface matched that of PDMS and lacked characteristic functional groups, such as nitrogen‐containing groups, present in the hydrogel layer. These results indicate that the failure primarily occurs within the substrate rather than at the interface. The same applies to stainless steel and ZrO_2_ plates, as shown in Figure S16 (Supporting Information).

The experiment further investigated the effects of temperature and humidity on the adhesion strength between the hydrogel layer and glass. Given that the typical operating temperature of hydrogel layers does not exceed 100 °C, we selected 75 °C as the test temperature. At this temperature, the normal adhesive strength between the hydrogel layer and the glass surface was measured to be 3.44 ± 0.60 MPa. This value is comparable to the normal adhesive strength (3.51 ± 0.35 MPa) and the shear adhesive strength (3.48 ± 0.62 MPa) measured at room temperature, indicating that the system can maintain a relatively high adhesive strength even under elevated thermal conditions. In addition, the adhesive strength under a high‐humidity environment (80–90% relative humidity for 2 h, 21 °C) was 3.56 ± 0.92 MPa, which is similar to the value obtained under ambient conditions (30–40% relative humidity, 21 °C). These results suggest that, within a short exposure period, humidity has a negligible effect on the adhesion performance of the hydrogel layer.

In addition to the polyacrylamide (PAAm) based hydrogel layer on PDMS mentioned above, we have recently found that polyacrylic acid (PAA) and poly(N,N‐dimethyl acrylamide) (PDMAA) based hydrogel layers on PDMS substrates also exhibit strong adhesion when in contact with glass surfaces (as shown in Figure S17, Supporting Information). Such hydrogel coatings hold promise for enhancing the adhesion strength of PDMS, thereby improving its performance in applications such as object gripping and fixation during manufacturing and transportation processes.

## 3 Conclusion

This study has demonstrated that the hydrogel layer exhibits exceptionally high friction and adhesion forces during the transition from a wet to a dry state, in contrast to the low friction observed in fully dry or water‐lubricated conditions. The COF between the hydrogel layer and a ZrO_2_ ball can reach up to 57.5 during this transition, highlighting the unique adhesive properties of hydrogels in this phase. These properties enable the hydrogel to effectively grasp objects on a variety of surfaces, including smooth, rough, and even fibrous textures. Notably, the hydrogel layer with shrinkage‐induced microstructures exhibits a high adhesion strength of 3.48 MPa on glass and 3.64 MPa on Si substrates.

The strong adhesion observed in this study is attributed to the formation of microstructures during the dehydration of the hydrogel layer, as well as the intermolecular interactions between the hydrogel layer and objects. These findings challenge the traditional view of hydrogels as primarily lubricating materials and provide new insights into their functional capabilities, particularly in applications requiring active gripping and adhesion. Beyond expanding our understanding of hydrogel adhesion mechanisms, this research opens new possibilities for their use in biomedical devices, soft robotics, and advanced gripping technologies. Future work could focus on optimizing these properties for specific industrial and medical applications, further enhancing the versatility of hydrogel‐based systems.

## 4 Experimental Section

### Fabrication of Hydrogel Layer on PDMS

The PAAm hydrogel layer on PDMS, fabricated using a Sylgard 184 (Dow Corning) mixture, was prepared through a series of sequential steps. Initially, the PDMS substrates were cleaned with isopropanol and DI water, followed by drying under an air stream. Subsequently, the substrates were exposed to ultraviolet (UV) light for 5 min. The UV‐treated substrates were then immersed in an ethanol solution containing 10 wt.% benzophenone (99%, Sigma–Aldrich) for 5 min. After gentle rinsing with isopropanol and air drying, the substrates were placed in an aqueous solution composed of 30 wt.% acrylamide (98+%, Thermo Scientific) with 1 wt.% 2‐Hydroxy‐4’‐(2‐hydroxyethoxy)‐2‐methylpropiophenone (Irgacure‐2959, 98%, Aldrich). The monomer solution was irradiated with UV light for 55 min. Following hydrogel formation, unreacted reagents were thoroughly removed by extensive rinsing with DI water for 24 h, after which the samples were air‐dried at room temperature, yielding a dry hydrogel layer on PDMS. To obtain a wet hydrogel layer, the dried hydrogel was either submerged in DI water or had a few drops of water applied to its surface, enabling rapid water absorption and transition to a wet state within seconds. Excess surface water was gently wiped away using non‐woven fabric.

Following the steps outlined above, we can prepare a hydrogel layer on one side of the PDMS substrate. This was because the density of the PDMS substrate was lower than that of water, causing it to float in the aqueous solution, allowing the submerged portion to form a coating. To achieve a hydrogel coating on both sides, the PDMS substrate must be fully immersed in the solution, which can be accomplished by attaching a weight (such as a transparent glass) to ensure complete submersion.

The preparation process for the PAA or PDMAA hydrogel layer on PDMS substrates was generally consistent with that of the PAAm layer. The main differences were mentioned as follows. For the preparation of the PAA hydrogel layer, the PDMS samples were first immersed in an isopropanol solution containing 10 wt.% benzophenone for 5 min. After gentle rinsing with isopropanol and air drying, the substrates were placed in an aqueous solution composed of 10–30 wt.% acrylic acid (98%, extra pure, stabilized, Thermo Scientific Chemicals) with a small amount of 2‐Hydroxy‐2‐methylpropiophenone (1173, 96.0+%, TCI America™) as the photoinitiator. For the preparation of the PDMAA hydrogel layer, the PDMS samples were first immersed in an ethanol solution containing 10 wt.% benzophenone for 5 min. After gentle rinsing with isopropanol and air drying, the substrates were placed in an aqueous solution composed of 10–30 wt.% N,N‐dimethylacrylamide (99%, Sigma–Aldrich) with a small amount of 2‐Hydroxy‐2‐methylpropiophenone as the photoinitiator. After the above steps, the monomer solution was irradiated with UV light for 55 min.

### Tribological Performance Tests

The friction behaviors of the hydrogel layer coated PDMS were carried out using a tribometer (Anton Paar, Austria) at room temperature. ZrO_2_, 304 stainless steel, or nylon balls with a diameter of 2 mm were used as the upper friction pair. For the reciprocating friction experiment, the applied load was 5 mN, the reciprocating amplitude was 1 mm, and the velocity was set to 8, 6, 4, 2, 1, or 0.5 mm s^−1^. For the single sliding friction experiments, the applied load was 1, 2, 3, 4, or 5 mN, the sliding velocity varied from 3 to 30 µm s^−1^, and the maximum sliding stroke was set to 5 mm. The experiments were carried out under room temperature conditions (≈21 °C) and ambient relative humidity (30–40%).

### AFM tests of the Hydrogel Layer

The force‐displacement curves of the hydrogel layer in its dry state, wet state, and during the transition from wet to dry were obtained through measurements using an AFM (Oxford Instruments, US). An antimony‐doped silicon tip (Bruker, US) was used in the AFM tests. The force constant of the cantilever was 42 N/m.

### Rheological Behavior and Modulus Tests

The rheological tests of hydrogel layer on the PDMS substrate were conducted using a rheometer (AR‐G2, TA Instruments, USA) with a 20 mm stainless steel plate at a frequency of 1.0 Hz, a duration of 100 s, and a temperature of 21°C (room temperature), with a strain of 1.0%. Considering the tight contact between the stainless steel plate and the hydrogel layer during testing, the evaporation of water from the hydrogel layer occurs very slowly. To account for this, we recorded the duration between tests, i.e., the time elapsed after the stainless‐steel plate was lifted, as the dehydration time of the hydrogel. For example, if the test times were T1, T3, T5, etc., and the interval times were T2, T4, T6, etc., then the time values in Figure 3b,c refer to the cumulative duration of T2, T4, T6, and so on. Due to the influence of environmental factors on the dehydration process during testing, the dehydration process observed in this test did not entirely correspond to that occurring during the friction tests.

### Adhesion Force Tests

Mechanical Characterizations. The adhesion force tests on the hydrogel layer coated PDMS and other objects (including stainless steel, ZrO_2_ plate, glass plate, non‐woven fabric) were conducted using a tensile testing machine (Shimadzu, Japan). For the adhesion force tests shown in Figure 3d–f, the applied pressure hold value was set to 10 N for a duration of 100 s. The maximum lifting height was 2 mm, with a speed of 10 mm min^−1^, and the dwell time at the highest point was 0 s.

For the shear adhesion strength tests in Figure 5, the dimensions of the double‐layer hydrogel sample between the glass plates were ≈4.00 × 2.50 × 0.45 mm. Before the test, the loading force between the double‐sided hydrogel layer and glass plates was 0.25, 2, 3, and 5 N. The stretching speed in the test was 20 mm min^−1^. For the test in Figure S12 (Supporting Information), the contact area between the hydrogel layer and the nonwoven fabric was ~8.85 × 3.49 mm. Before conducting the adhesion experiments, we cleaned and treated the surfaces of Si wafers, glass plates, and ZrO₂ plates with UV/Ozone cleaner (Bioforce Nanosciences, US) to remove surface contaminants. These materials were then brought into contact with the wetted hydrogel layer under a force of 0.25–5 N and left in air for ≈24 h before performing the adhesion tests. Normal adhesion testing procedures were similar to the shear adhesion test described above. In addition, the effects of different temperatures and ambient humidity levels on the adhesive strength of the hydrogel layer were investigated. The temperature ranged from 21 °C to 75 °C, and the ambient humidity was controlled using a humidifier (T‐326, Conghui Co., Ltd, China) to maintain levels between 30% and 90%. All adhesion test results were reported as the average values of at least three independent measurements.

### Microstructure Observation of the Hydrogel Layer

The surface morphologies of the hydrogel layer in the dry state, wet state, and during the wet‐to‐dry transition were examined using a microscope (ZEISS, Germany). The cross‐sectional morphology of the hydrogel layer was also analyzed with the same microscope, both before and after water absorption. Furthermore, the interfacial interactions between the hydrogel layer and various grasped objects, along with the water absorption process, were investigated under the microscope. To better visualize the contact interface, the microstructures were observed from the hydrogel layer‐PDMS side, owing to its relatively high transparency. The cross‐sectional morphologies of the hydrogel layer on PDMS were characterized using cryo‐scanning electron microscopy (Cryo‐SEM, Hitachi Su8600, Japan).

## Conflict of Interest

The authors declare no conflict of interest.

## Author Contributions

The manuscript was written through the contributions of all authors. All authors have given approval to the final version of the manuscript.

## Supporting information



Supporting Information

Supplemental Movie 1

## Data Availability

The data that support the findings of this study are available from the corresponding author upon reasonable request.
