# Supplementary material for: Ultra‐High Friction and Adhesion in Hydrogel Layer Driven by Wet‐to‐Dry Transition Dynamics
Source: Adv Sci (Weinh). 2025 Jul 12;13(15):e07827. doi: 10.1002/advs.202507827 (PMC13042388; doi:10.1002/advs.202507827)
Supplement: Supplementary file 1 — Supporting Information [file ADVS-13-e07827-s002.docx]

Supporting Information

Ultra-High Friction and Adhesion in Hydrogel Layer Driven by Wet-to-Dry Transition Dynamics

Chenxu Liu, Tianhui Sun, Wenqing Chen, Hongjian Zhang, Pan Huang, Lin Yang, Yuan Yao, Qiongyao Peng, Ying Hu, Yonggang Meng, Yu Tian*, Hongbo Zeng*

C. Liu, H. Zhang, P. Huang, L. Yang, Y. Yao, Q. Peng, H. Zeng

Department of Chemical and Materials Engineering, University of Alberta, Edmonton T6G 1H9, Canada

E-mail: [hongbo.zeng@ualberta.ca](mailto:hongbo.zeng@ualberta.ca)

C. Liu, T. Sun, W. Chen, Y. Meng, Y. Tian

State Key Laboratory of Tribology in Advanced Equipment, Tsinghua University, Beijing 100084, China

E-mail: [tianyu@mail.tsinghua.edu.cn](mailto:tianyu@mail.tsinghua.edu.cn)

Y. Hu

Heavy Machinery Engineering Research Center of Education Ministry, Taiyuan University of Science and Technology, Taiyuan 030024, China


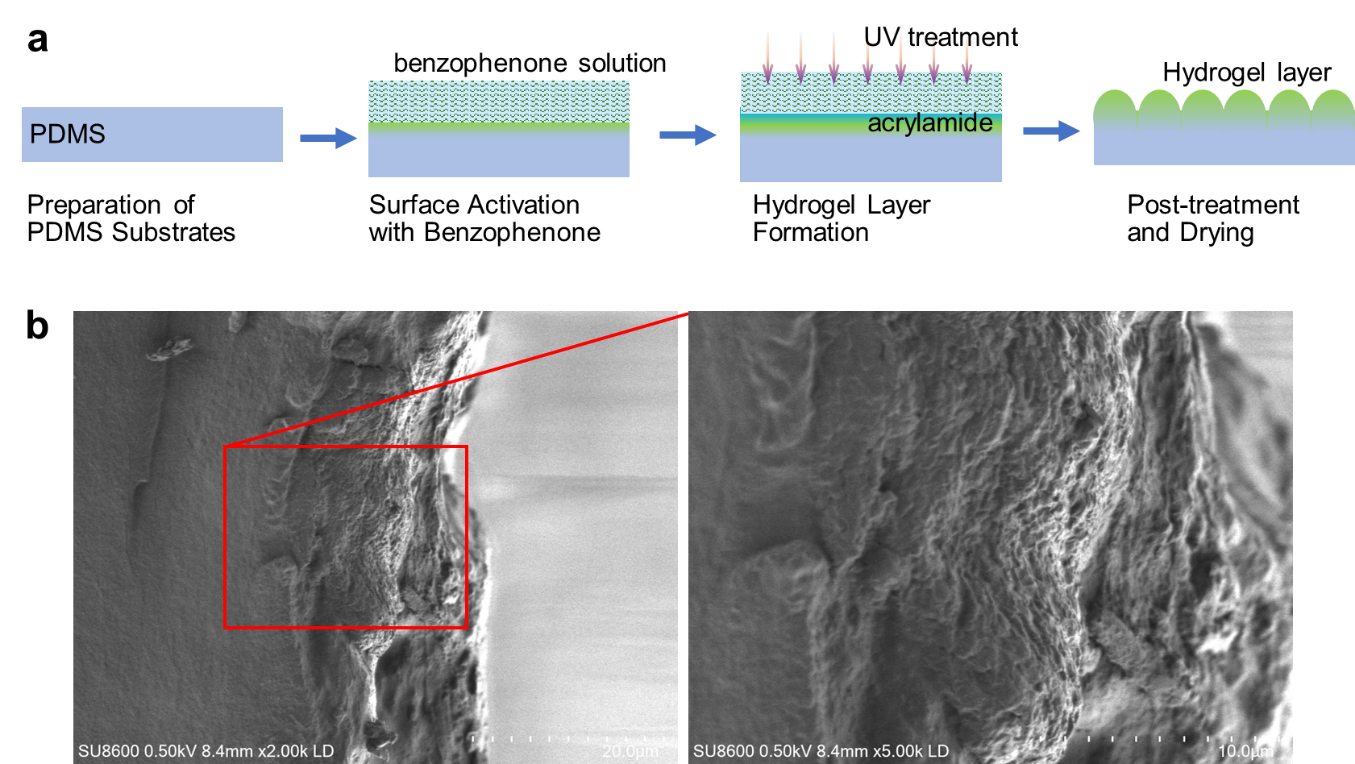


**Figure S1.** (a) Schematic illustration of the hydrogel layer preparation process; (b) Cryo-scanning electron microscopy (Cryo-SEM) observation of the thickness of the hydrogel layer.


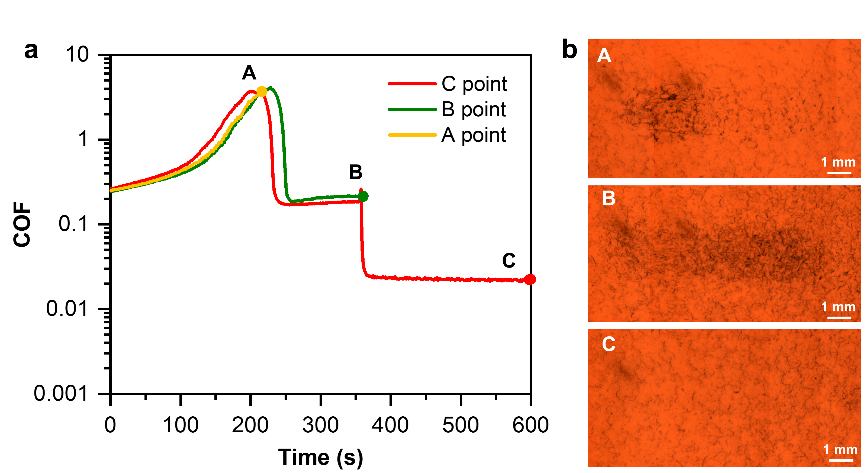


**Figure S2.** COFs and wear morphology microscopic observations of friction area on the hydrogel layer at different friction stages: (a) Variation of the COF over time: from 0 s to point A represents the drying process of the wet hydrogel; from point A to point B corresponds to the friction stage of the dried hydrogel; and from point B to point C indicates the lubrication stage after water is introduced as the lubricant; (b) Microscopic morphologies of the lower friction pair at the end points of the three stages described above. The experiment was conducted at room temperature, with a normal load of 5 mN and a sliding speed of 2 mm/s.


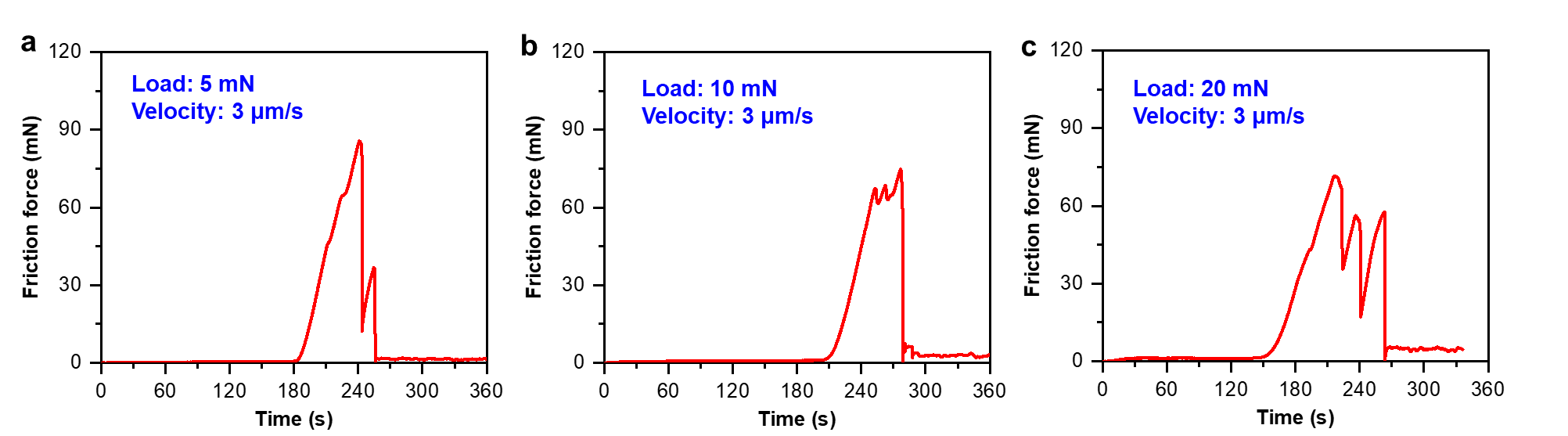


**Figure S3.** Friction force variation over time under different loads: (a) Load of 5 mN, velocity of 3 μm/s; (b) Load of 10 mN, velocity of 3 μm/s; (c) Load of 20 mN, velocity of 3 μm/s.


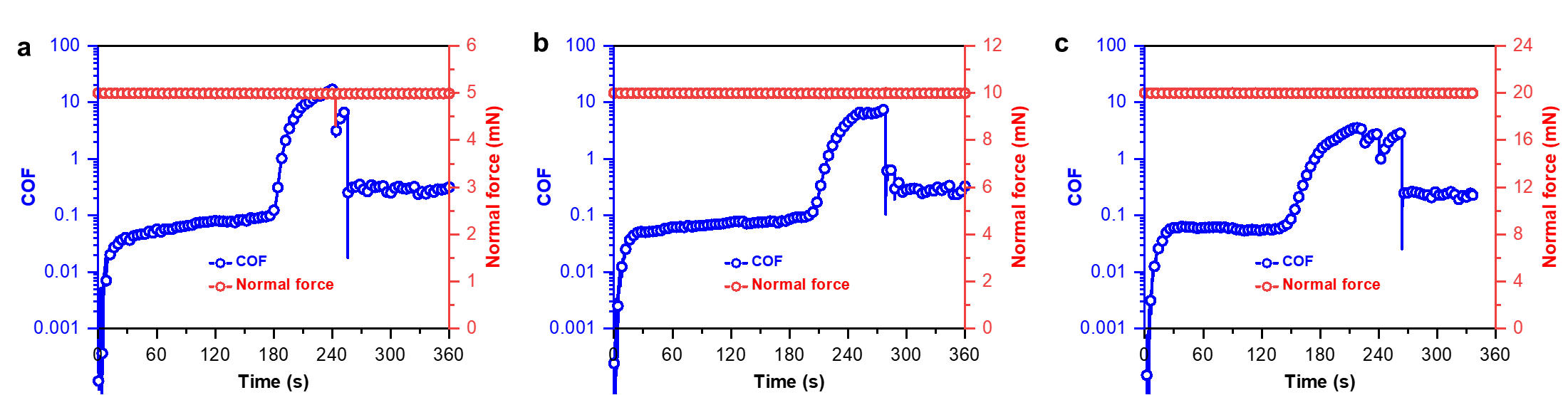


**Figure S4.** COF and normal force variation over time under different loads: (a) Load of 5 mN, velocity of 3 μm/s; (b) Load of 10 mN, velocity of 3 μm/s; (c) Load of 20 mN, velocity of 3 μm/s.


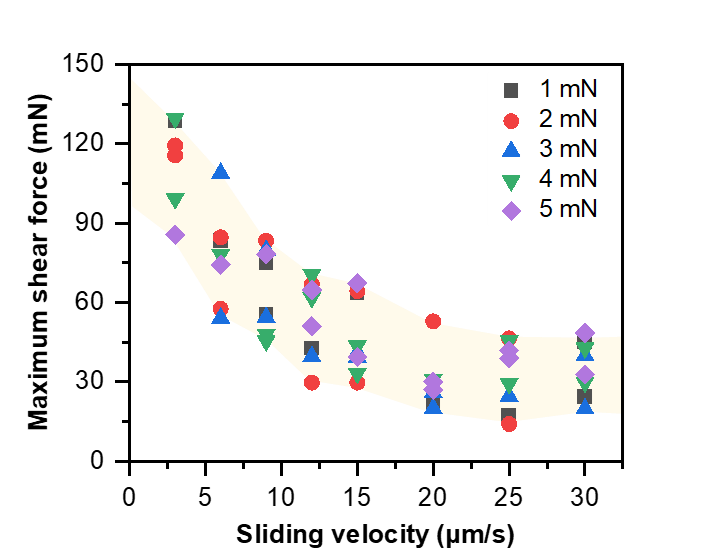


**Figure S5.** Maximum friction force at different loads and velocities. In these experiments, the upper friction pair was a ball with a diameter of 2 mm, the applied load ranged from 1 to 5 mN, the sliding velocity varied from 3 to 30 μm/s, and the maximum sliding stroke was set to 5 mm.


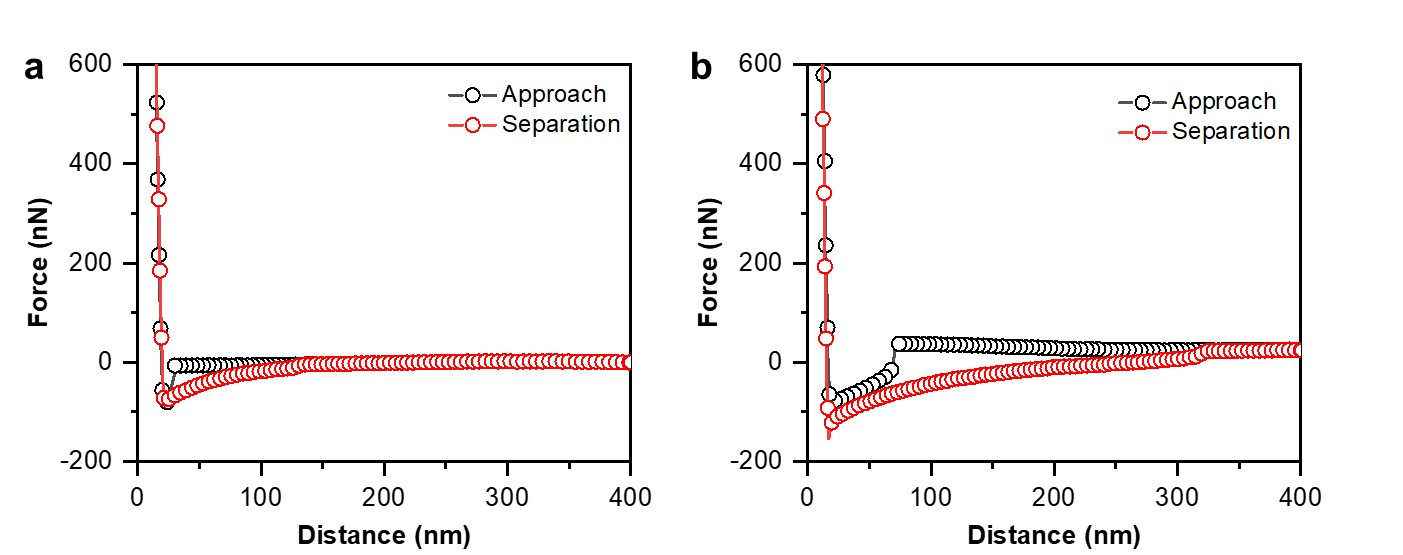


**Figure S6.** (a) AFM-measured force-distance curve of the hydrogel layer in the dry state; (b) AFM-measured force-distance curve of the hydrogel layer in the wet state or covering the transition. The force constant of the cantilever was 42 N/m.


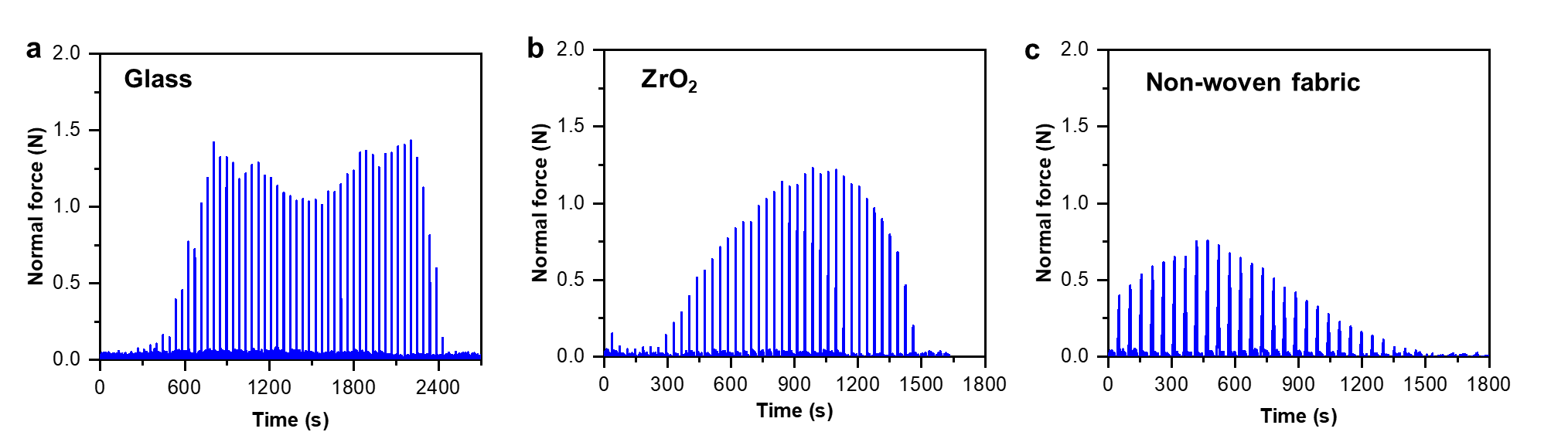


**Figure S7.** The variation of the adhesion force between the hydrogel layer and different plate over time: (a) Glass; (b) ZrO_2_; (c) Non-woven fabric.


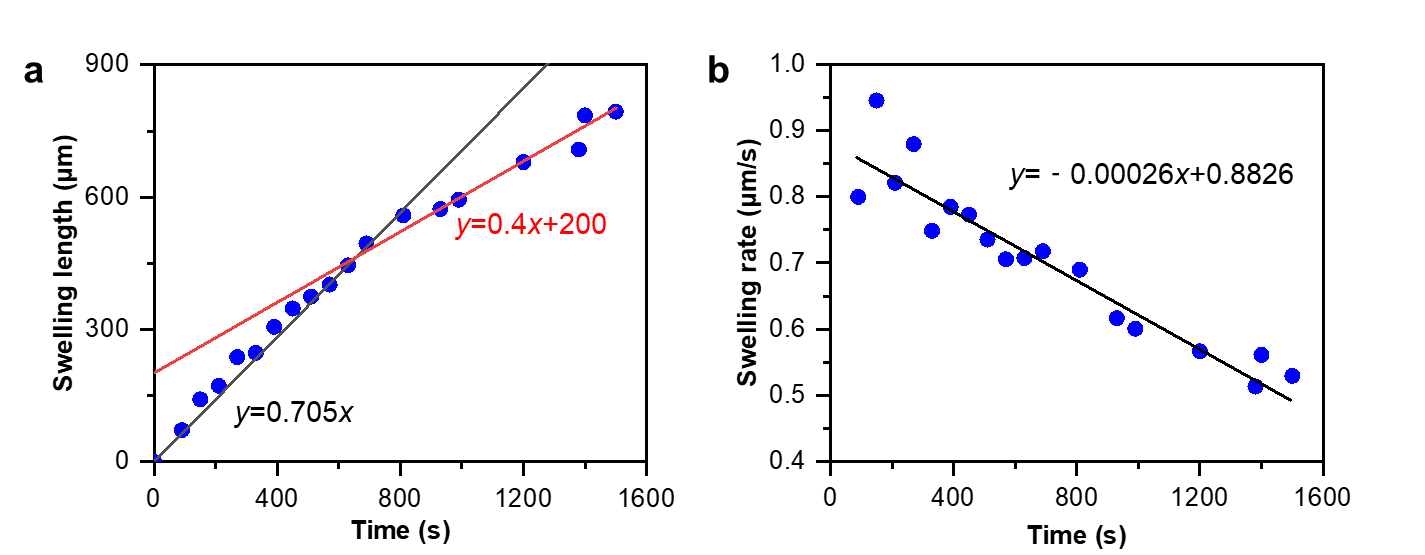


**Figure S8.** The relationship between the swelling length (a) and rate (b) of the hydrogel layer at the hydrogel-stainless steel interface over time. According to (b), the adsorption rate decreases linearly at 0.00026 μm/s^2^.


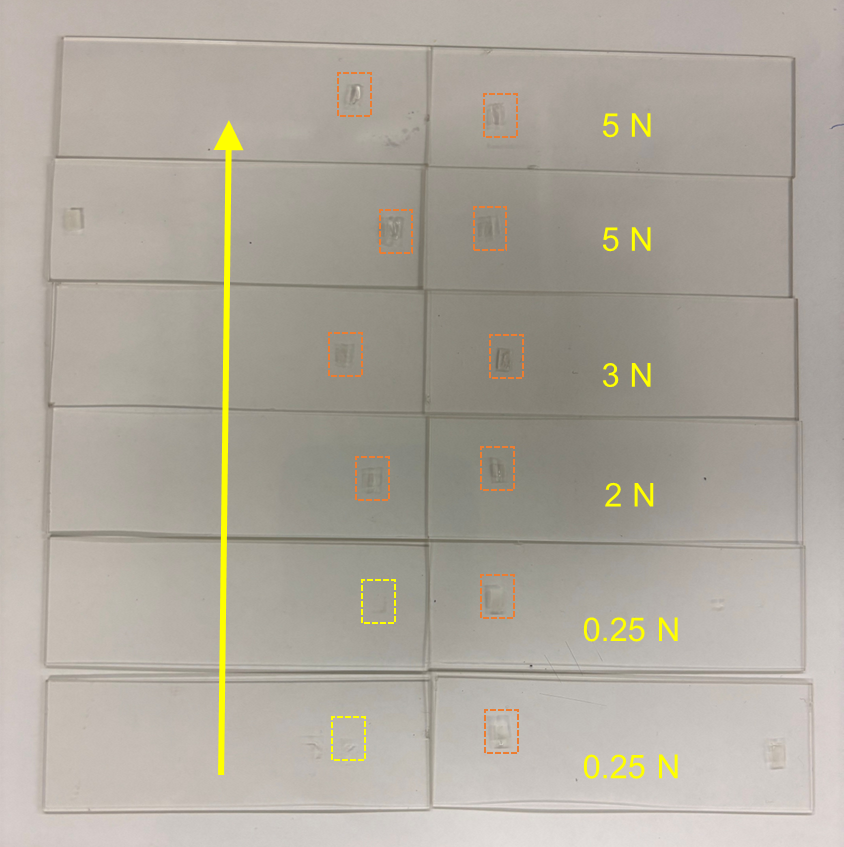


**Figure S9.** Residual morphology of the double-layer hydrogel layer on two glass plates after adhesion testing of samples prepared under different loading forces. As the applied load increases, a greater amount of the sample remains on both glass surfaces, indicating a larger contact area between the hydrogel layer and the glass, leading to higher shear strength. The size of each glass in the photos is 25×75×1 mm.

**Figure S10.** The relationship between the shear strength and displacement measured in adhesion test between the commercial double-sided tapes and glass plates, with a stretching speed of 20 mm/min.


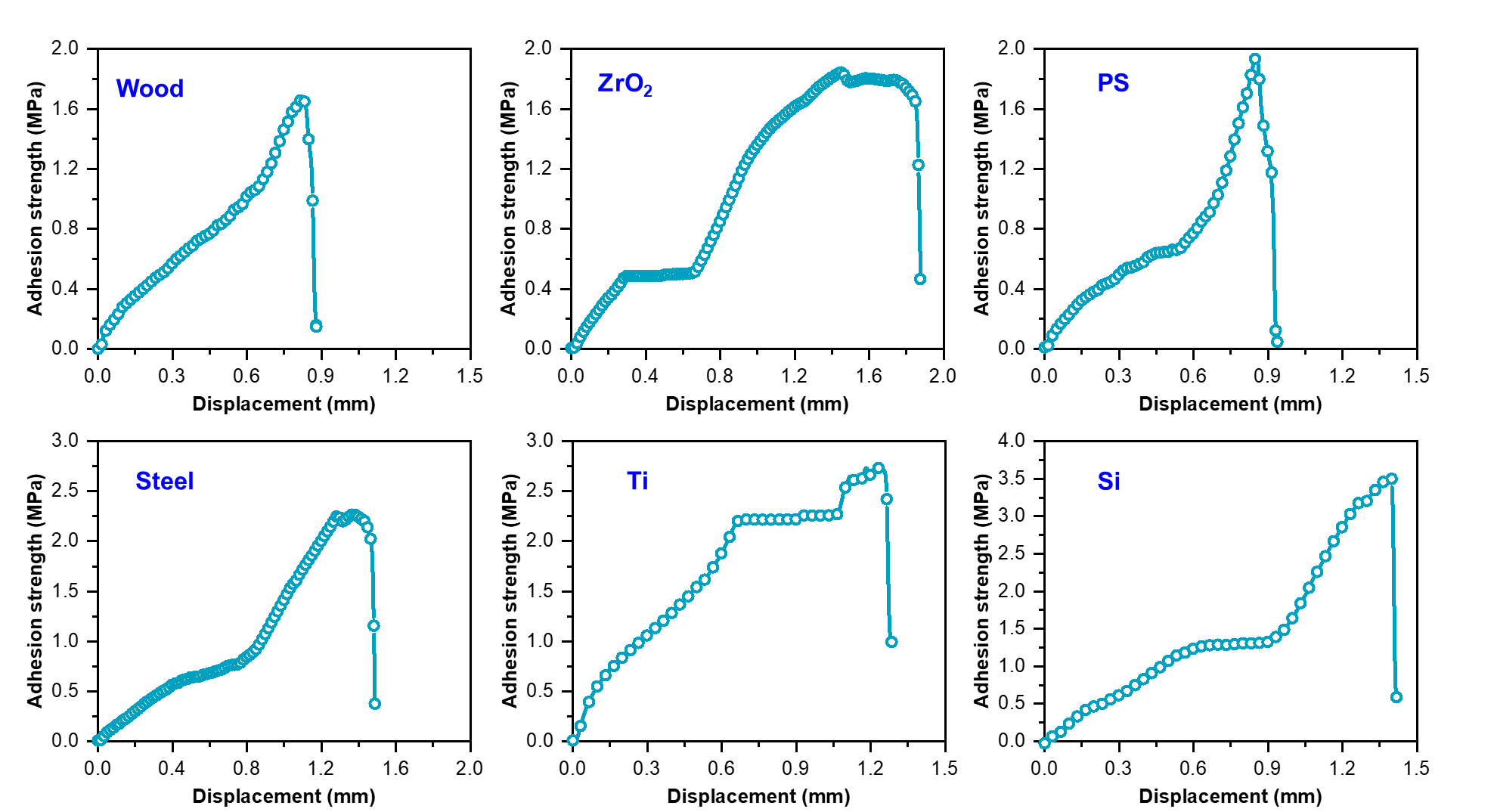


**Figure S11.** The relationship between the shear strength and displacement measured in adhesion test between the double-sided hydrogel layer and wood, ZrO_2_, PS, stainless steel, Ti, Si wafer, with a stretching speed of 20 mm/min.


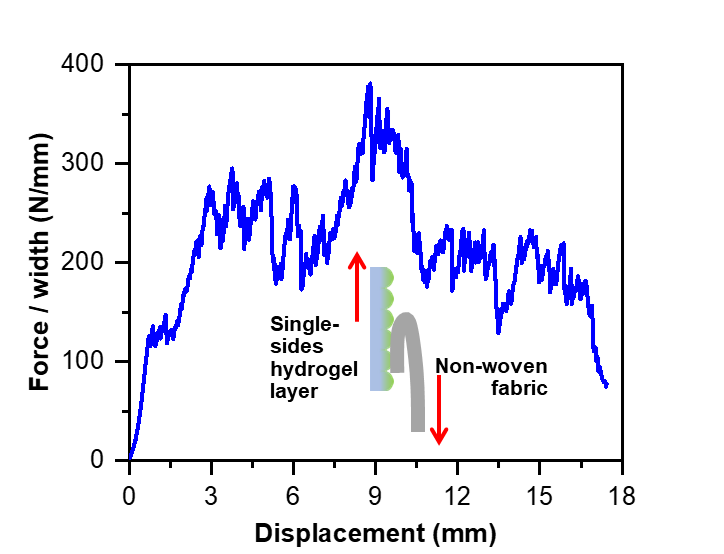


**Figure S12.** The relationship between the peeling force per unit width and displacement measured in a 180° peeling test between the hydrogel layer and nonwoven fabric, with a stretching speed of 20 mm/min.


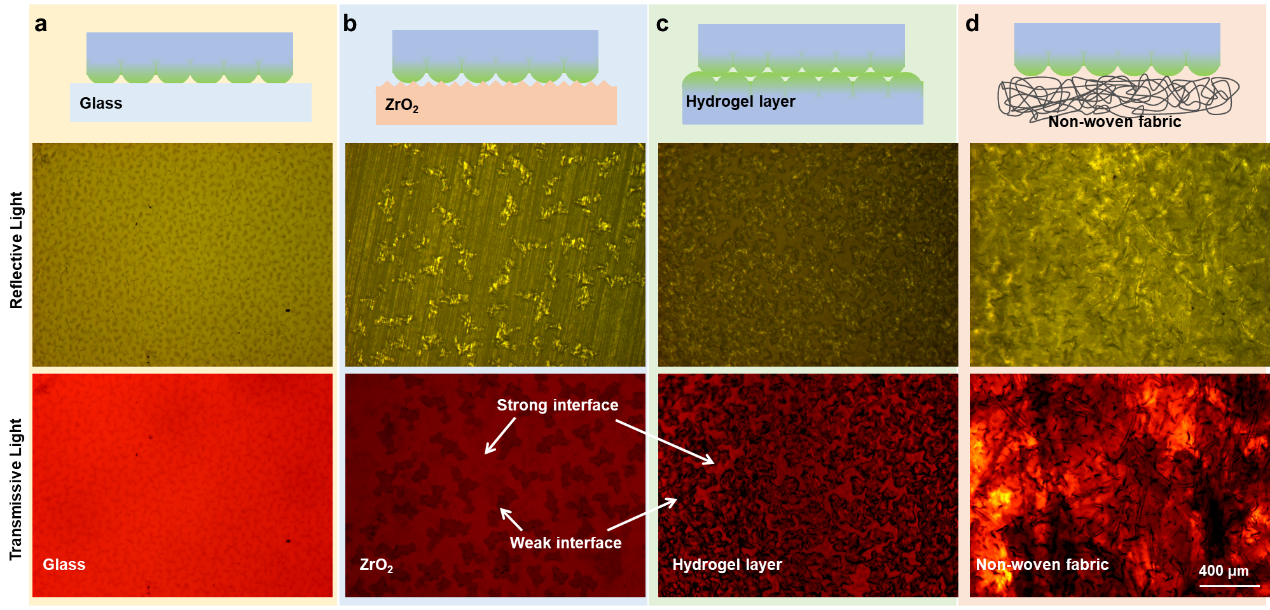


**Figure S13.** Schematic diagram and microscopic observation images of the adhesion interface between the hydrogel layer and different objects captured using reflected and transmitted light: (a) Glass plate; (b) ZrO_2_ plate; (c) Hydrogel layer coated PDMS plate; (d) Non-woven fabric.


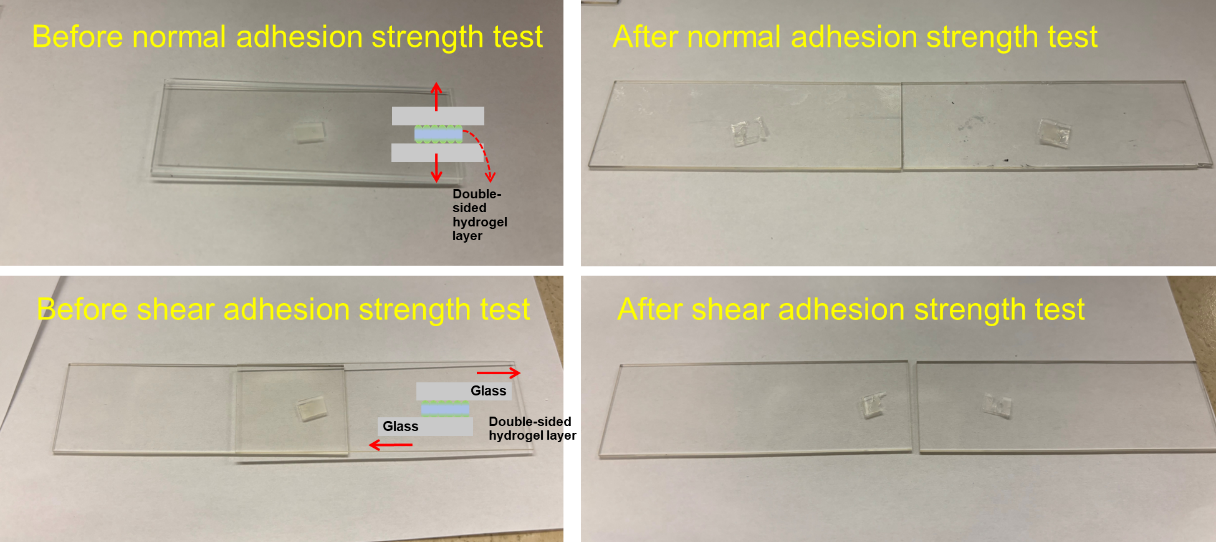


**Figure S14.** Photos of the samples before and after the mechanical performance tests. The size of each glass in the photos is 25×75×1 mm. From the figures, it can be seen that after both the shear adhesion strength and normal adhesion strength tests, the failure did not occur at the interface between the hydrogel layer and the gripped object but rather within the substrate (PDMS) itself.


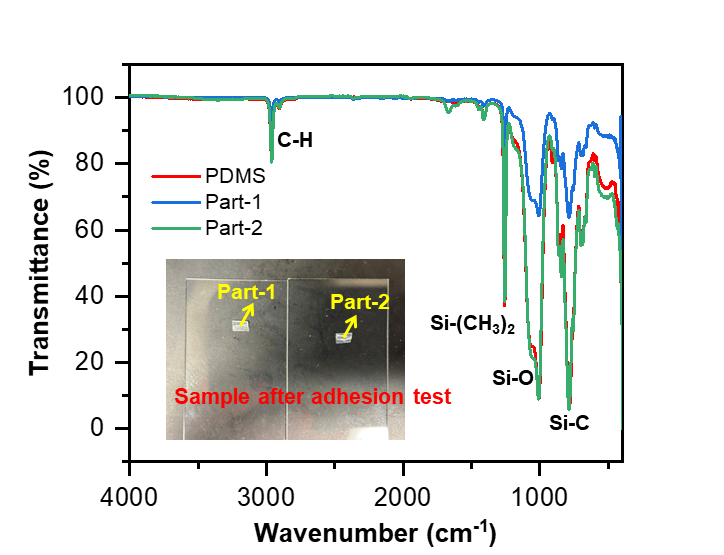


**Figure S15.** FT-IR spectra of the residual sample after the shear adhesion test between the double-layer hydrogel layer and the glass, compared with the FT-IR spectrum of bare PDMS. The inset shows the actual morphology of the residual sample (sample size approximately 3.5 × 2 cm).


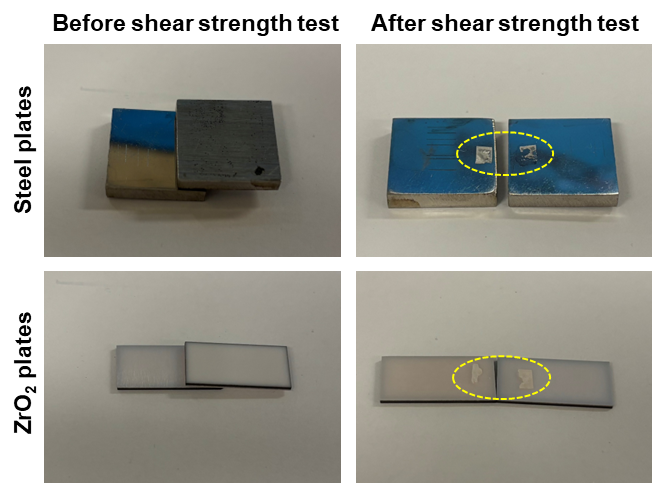


**Figure S16.** Photos of the samples before and after the shear strength tests. The size of each steel plate and ZrO_2_ plates in the photos is 20×20×3 mm and 20×10×1 mm, respectively.


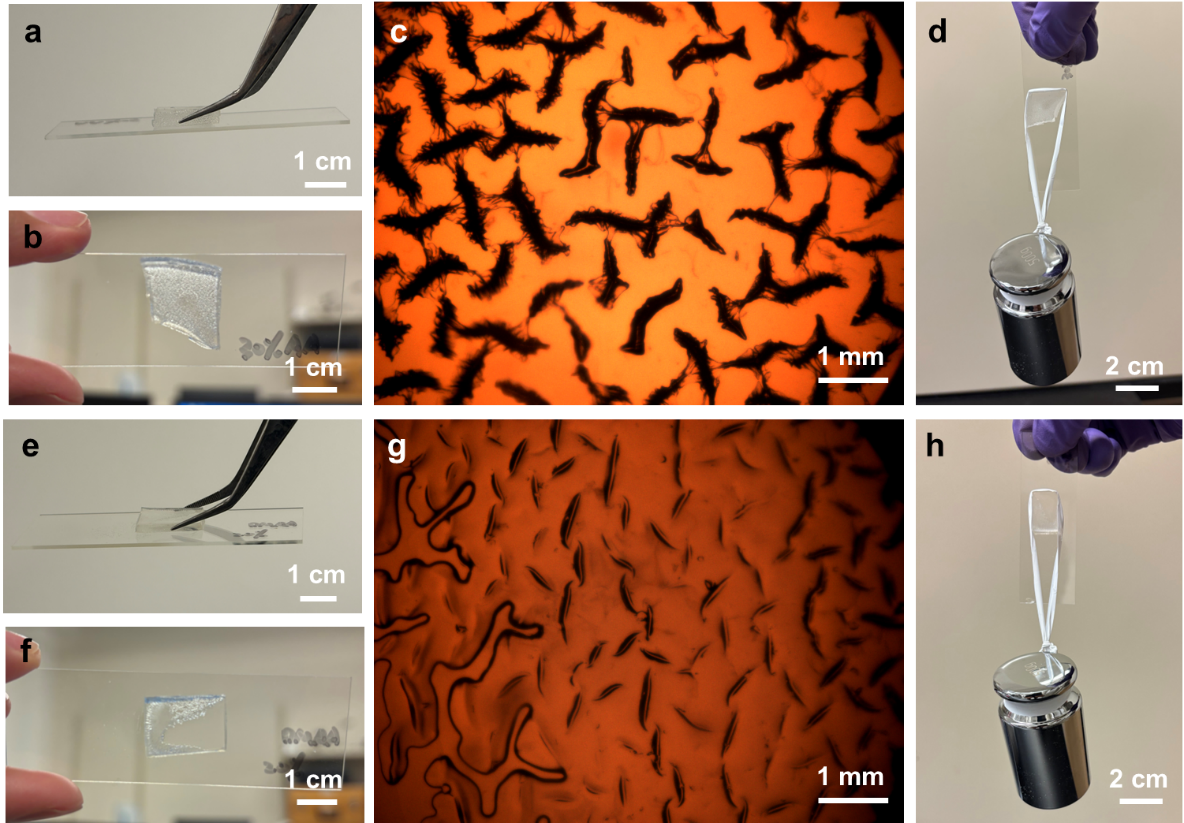


**Figure S17.** Photographs, microscopic morphology, and demonstration images of different hydrogel layers in contact with glass and lifting a 500 g weight: (a–d) show the hydrogel layer prepared using 30% acrylic acid (AA) as the monomer, while (e–h) show the hydrogel layer prepared using 20% N,N-dimethyl acrylamide (DMAA) as the monomer.
